# Recurrent Sinus Pauses: An Atypical Presentation of Temporal Lobe Epilepsy

**DOI:** 10.1155/2014/918247

**Published:** 2014-09-28

**Authors:** Martin Miguel Amor, Sherif Ali Eltawansy, Jeffrey Osofsky, Neil Holland

**Affiliations:** ^1^Department of Internal Medicine, Monmouth Medical Center, Long Branch, NJ 07740, USA; ^2^Section of Cardiology, Monmouth Medical Center, Long Branch, NJ 07740, USA; ^3^Section of Neurology, Monmouth Medical Center, Long Branch, NJ 07740, USA

## Abstract

Autonomic dysfunction related to seizures may give rise to a broad spectrum of cardiovascular abnormalities. Among these, ictal bradycardia and conduction delays may be encountered. Failure to recognize these abnormalities may contribute to sudden, unexplained death in epilepsy patients. We report a case of a Haitian female with temporal lobe epilepsy associated with recurrent sinus pauses.

## 1. Introduction

Epilepsies are known to alter autonomic function during the ictal, postictal, and interictal periods. Autonomic function at both sympathetic and parasympathetic levels may be affected. These effects may give rise to a broad spectrum of cardiovascular abnormalities. We report a case of a Haitian female with temporal lobe epilepsy associated with recurrent sinus pauses.

## 2. Case Report

**Figure 1:**
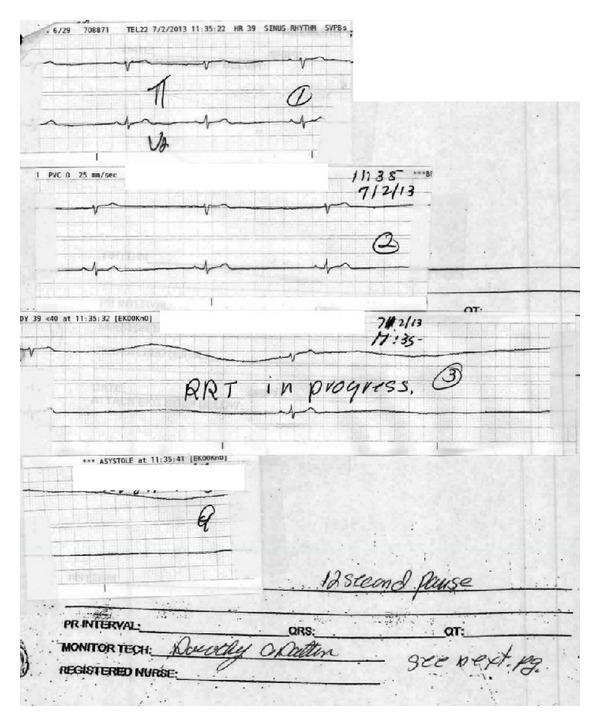
Telemetry tracing during the seizure episode, revealing sinus bradycardia, which eventually progressed to a 12-second pause.

**Figure 2:**
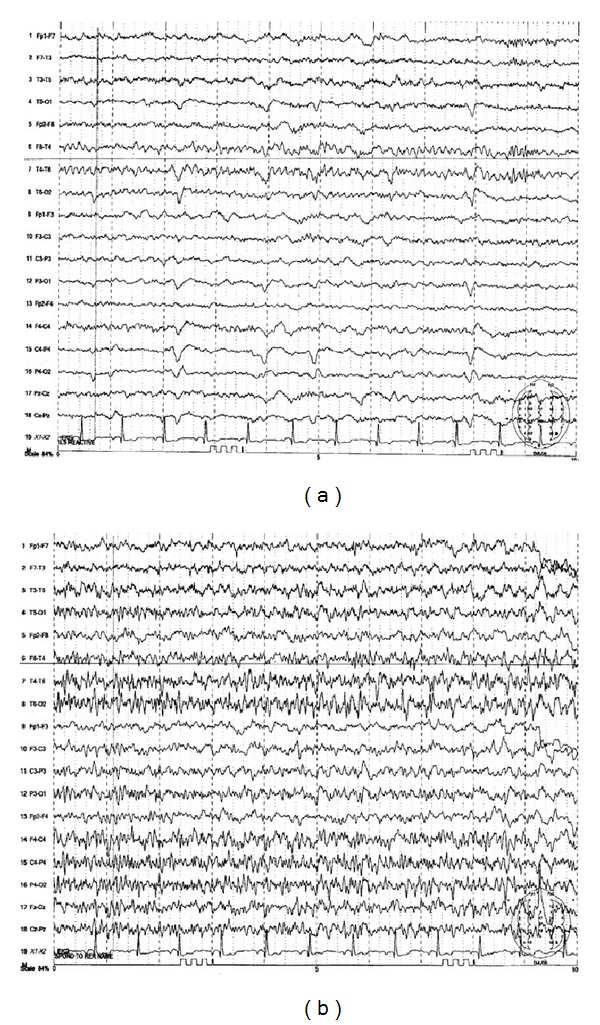
EEG revealing evolution of seizure, beginning with right temporal periodic lateralized epileptiform discharges (PLEDs) and then right temporal rhythmic sharp waves (a), followed by more widespread epileptiform activity (b).

**Figure 3:**
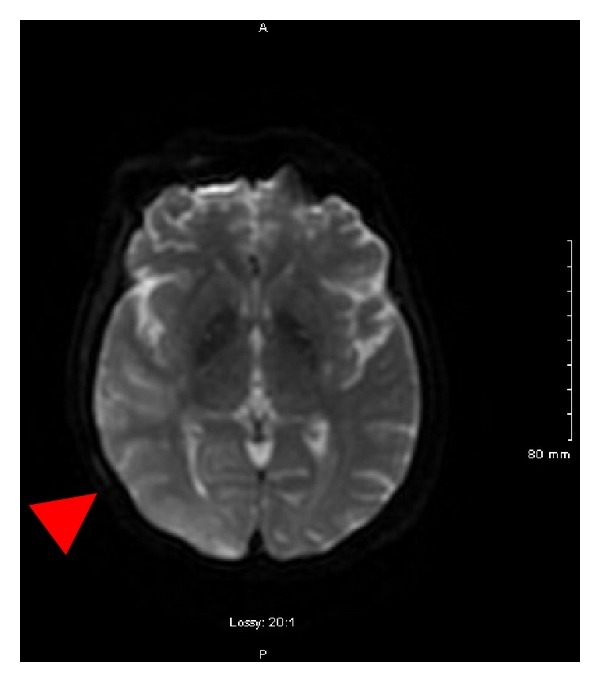
MRI of the brain showing a large area of gyral edema, sulcal effacement, and cortically based diffusion restriction involving the right occipital lobe and right posterior temporal and parietal lobes (indicated by RED arrow).

A 58-year-old Haitian female with known history of hypertension was admitted for severe bilateral lower extremity weakness. CT scan of the thoracolumbar spine revealed severe kyphosis of T10-T11 secondary to anterior collapse of the T11 vertebral body. She received intravenous steroids and was evaluated for surgical intervention. She eventually underwent T11 corpectomy, fusion of T10-T12, implantation of biomechanical device at T11, anterior instrumentation of T10-12, and posterolateral fusion of T8-L3. Bone biopsy revealed evidence of osteomyelitis. She was started on a 42-day course of antibiotics. After surgery, she was noted to be increasingly lethargic and confused. Rapid response was called when she developed a complex partial seizure with secondary generalization. The seizure was terminated upon administration of intravenous Ativan. She was given a 1500 mg loading dose of Keppra followed by 500 mg twice daily maintenance dose. EKG monitoring during the seizure episode revealed sinus bradycardia, which eventually progressed to a 10-second sinus pause, approximately 20 seconds after seizure onset (Figure 1). She had 2 more similar seizure episodes during the same day. In each seizure episode, she would develop sinus bradycardia, followed by sinus pauses a few seconds after seizure onset. Interictal EKGs revealed normal sinus rhythm. She was started on a dopamine infusion and transferred to the ICU. EEG revealed periodic lateralized epileptiform discharges and a single seizure emanating from the right posterior temporal region (Figure 2). The seizure observed during the EEG focally originated from the T6 area and then had secondary generalization. It lasted around 75 seconds and clinically manifested as blank staring. MRI revealed a large area of gyral edema, sulcal effacement, and cortically based diffusion restriction involving the right occipital lobe and right posterior temporal and parietal lobes (Figure 3). Lumbar tap revealed normal findings. She did not have any further seizure episodes. On the succeeding hospital day, she underwent DDD pacemaker insertion and did not develop any more pauses. Repeat EEG revealed no lateralizing or epileptiform discharges. Her prolonged hospital course was complicated by hemorrhagic pleural effusion, venous air embolism after central line removal, and surgical site infection. These complications were treated accordingly. She was discharged to an acute rehabilitation facility after 26 days of hospital stay.

## 3. Discussion

Epileptiform activity from the amygdala, anterior cingular cortex, and insula of the temporal lobe can produce cardiac rate and rhythm abnormalities [1–3]. Such changes may contribute to seizure-induced cardiovascular dysfunction, which in turn, can lead to sudden, unexplained death in epilepsy.

Changes in heart rate are frequently observed during seizure episodes [4]. Ictal heart rate increase is more common and can precede EEG seizure onset by approximately 13 seconds in temporal lobe epilepsy [5]. Ictal bradycardia, on the other hand, occurs in fewer than 2% of seizures, is usually of frontal or temporal lobe origin, and may be more frequent in males and in patients with left-sided epileptiform foci. Seizure-induced asystole is rare and is usually associated with preexisting cardiac disorders [6, 7]. This is usually preceded by sinus pauses and sinus bradycardia. This phenomenon is thought to be of central origin, mediated by vagal cardiac activation [8]. Our patient presented with frequent sinus pauses but did not progress into cardiac asystole.

There is no absolute agreement on laterality of seizure onset and degree of cardiovascular dysfunction. Oppenheimer et al. [9] demonstrated that stimulation of the left insular cortex produced bradycardia and hypotension, while stimulation of the right insular cortex produced tachycardia and hypertension. Opherk et al. [4], on the other hand, found that the hemisphere of origin, region of onset, and seizure duration did not influence ictal changes in heart rate. Our patient presented with sinus pauses and epileptiform activity that was localized to the right temporal lobe.

EKG rhythm abnormalities may occur in 35% of generalized seizures [1]. Arrhythmias documented during seizures included atrioventricular block, atrial fibrillation, supraventricular tachycardia, premature ventricular complexes, and bundle-branch blocks [2, 3]. Our patient, however, did not have these cardiac arrhythmias. Her interictal EKGs revealed normal sinus rhythm.

Ictal bradycardia is seen primarily in seizures involving the temporal lobe but may occur particularly with bilateral spread of seizure activity [10, 11]. Our patient presented with complex partial seizures with secondary generalization, associated with sinus pauses preceded by sinus bradycardia. The MRI, EEG, and EKG findings in our patient, therefore, demonstrate the vital role of the temporal lobe in cardiovascular autonomic regulation.

## 4. Conclusion

Temporal lobe epilepsy is associated with autonomic and cardiovascular dysfunction. This may take the form of changes in cardiac rate and rhythm. While increases in heart rate are more common, ictal bradycardia should also be carefully detected and addressed, to prevent progression into cardiac asystole. Early suspicion and recognition of these events may aid the clinician in averting sudden, unexplained death in epilepsy.
